# THE ELDER ABUSE HELPLINE FOR CONCERNED PERSONS (CPS): HOW SERVICES FOR CPS IDENTIFY AND MITIGATE FE AND ABUSE

**DOI:** 10.1093/geroni/igad104.0883

**Published:** 2023-12-21

**Authors:** Lisa Rachmuth

**Affiliations:** Weill Cornell Medicine, New York City, New York, United States

## Abstract

Elder abuse can profoundly impact older adults who experience it. Historically, little is known, however, about another group of individuals in the lives of older adults: non-abusing family members, friends, and neighbors, referred to as “Concerned Persons” (CPs). Through the Elder Abuse Helpline for Concerned Persons (the Helpline), a program at Weill Cornell Medicine, the CPs receive supportive counseling for themselves and referrals to ongoing services for themselves and the Older Adults who are abused. Financial exploitation and abuse often go unrecognized by many. Previous research on the first year of data from Helpline calls indicated that CPs might be critical in enabling support and help-seeking for elder abuse victims, and may be an essential intervention target group to help promote help-seeking behaviors of Older Adult Financially exploited victims. This presentation will highlight findings from a recent study involving secondary analysis of de-identified Helpline data that a) explored characteristics of CPs, alleged victims/abusers, as well as types and circumstances surrounding alleged abuse; and b) characterized the range of services and interventions requested and provided to concerned persons. Primary questions of interest included ones such as, “What are CP’s relationships to alleged victims/abusers?; How do the patterns, types, and circumstances surrounding alleged abuse vary across characteristics of CPs and their relationships to alleged victims/abusers?; What are the common concerns of CPs and the type of services requested by CPs?” Findings from this study improve our current understanding of CPs in the lives of alleged elder abuse victims and CPs’ help-seeking behaviors.

